# Independent Predictors of Erectile Dysfunction in Type 2 Diabetes Mellitus: Is It True What They Say about Risk Factors?

**DOI:** 10.5402/2012/502353

**Published:** 2012-08-27

**Authors:** Faranak Sharifi, Mohammad Asghari, Yahya Jaberi, Oveis Salehi, Fatemeh Mirzamohammadi

**Affiliations:** ^1^Clinical Endocrinology, Metabolic Diseases Research Center, Zanjan University of Medical Sciences, Zanjan 4513986745, Iran; ^2^Internal Medicine, Zanjan University of Medical Sciences, Zanjan, Iran; ^3^Clinical Cardiology, Zanjan University of Medical Sciences, Zanjan, Iran; ^4^Cardiovascular Research Center, Shiraz University of Medical Sciences, Shiraz, Iran; ^5^Metabolic Diseases Research Center, vali-e-asr Hospital, Zanjan University of Medical Sciences, Zanjan 6109678395, Iran

## Abstract

*Introduction*. The aim of this study was to evaluate the independent predictors of ED in adult men with type 2 diabetes mellitus (T2DM). *Methods*. We have recruited 200 T2DM patients referred to our center between March 1, 2009 and March 1, 2010. All the patients were scored with the International Index of Erectile Function (IIEF)-5 questionnaires. Contribution of age, body mass index (BMI), smoking, blood pressure, lipid profile, fasting plasma glucose (FPG), glycosylated hemoglobin (HbA1c), free testosterone concentration, and duration of diabetes to risk of ED were evaluated. *Results*. Of 200 men with T2DM, 59.5% had ED (95%CI: 52%–67%). A negative significant correlation was found between potency score and HbA1c (*r*: 0.20,*P*: 0.01), FPG (*r*: 0.17, *P*: 0.03) and SBP (*r*: 0.18, *P*: 0.02) but not between other risk factors such as lipid profile, BMI, and serum testosterone level. By using multivariate logistic regression analysis, we found out that the only two independent predictors of ED in these group of patients are age (OR: 2.8, *P*: 0.01), and taking calcium channel blockers (CCB) (OR: 4.1, *P*: 0.01). *Conclusions*. Aging and taking CCB were the only two major predictors for ED but surprisingly other metabolic or sexual covariates in this study did not have predictive value for ED risk in T2DM patients.

## 1. Introduction

Erectile dysfunction (ED) is described as a persistent inability (more than 6 months) to attain and maintain an erection sufficient to have satisfactory sexual performance [1]. Erectile dysfunction is a common occurrence in aged men especially in diabetic ones, who are affected with diabetic vasculopathy and neuropathy [2–4]. ED is associated with a reduced quality of life, and unfortunately occurs at an earlier age in diabetic patients in compare with the general population [5–8]. It may occur in the early stages of diabetes, and occasionally the chief complaint of a diabetic patient. Total cost for the treatment of patients suffering from ED in the United States is estimated to be $400 million, and approximately one fourth of that are paid for diabetes-related ED [9]. Detection of the factors that are associated with ED and particularly could predict the risk of ED in diabetic patients is important to better management of the disease. Also regarding the impact of ED on quality of life, early diagnosis and treatment of ED in such men is important and might prevent or delay its progression. The aim of our study was to evaluate the relation between ED and some metabolic factors in patients with T2DM and determine the risk factors of ED in men with T2DM in order to early eliminate those factors in these patients.

## 2. Methods

In this cross-sectional study, all the subjects were recruited from Diabetic center of Zanjan, in the north of Iran. This study was approved by the review board at the institution, and local ethical committee. All the subjects who participated gave written informed consent. The study population consisted of 200 men with type 2 diabetes, enrolled between March 1, 2009, and March 1, 2010. The inclusion criteria for participation were all patients referred with diabetes mellitus type 2 who aged more than 30 years. The patients with major depressive disorder, history of spinal or prostate surgery, and accompaniment with any secondary causes of impotency were excluded.

Assuming 50% prevalence of erectile dysfunction, the sample size with 80% power of the two-tail test at the 0.05 level of significance would be 206 cases.

All of the men were interviewed face to face using a standardized questionnaire (international index of erectile function (IIEF)-5) [10] to detect ED. The questionnaire evaluates five parameters of sexual function including, erectile function, orgasmic function, sexual desire, intercourse satisfaction, and overall satisfaction. Each item was rated on a 5-point scale. Men with a score of ≤21 (range, 5–25) were considered to have ED. Complete personal and medical history was taken for each patient. Risk factors such as age, smoking habits, and medications were assessed by direct questioning. Weight and height were measured and body mass index (BMI) was calculated. Systolic and diastolic blood pressure were measured. Men were considered hypertensive if they had a diastolic blood pressure (DBP) of ≥90 mmHg, or systolic blood pressure (SBP) of ≥140 mmHg. Subjects under antihypertensive medication were obviously considered hypertensive patients. Serum total cholesterol, triglyceride, and HDL-cholesterol were measured using enzymatic techniques. Fasting plasma glucose was measured and glycosylated hemoglobin (HbA1c) percentage was determined by ion-exchange method. Free testosterone concentrations were measured with electrochemiluminescence immunoassay (ECLIA) using commercial kits (DRG, Germany). All measurements were made under standard conditions by one technician and with the same device.

Demographic data was obtained by using descriptive methods. Because of abnormal distribution of the variables, we compared them between ED positive and ED negative group with nonparametric Mann-whitney test. Correlation between potency score and each variable was analyzed using Pearson's correlation test. Odds ratios of ED associated with the predictor variables were computed using univariate and multivariate logistic regression. *P* < 0.05 was considered to indicate statistical significance. All the statistical analysis was performed by using SPSS ver.13 software.

## 3. Results

Of 214 men with type 2 DM, 9 refused to participate and 5 of them were not eligible for the study and were excluded. A total of 200 patients were finally enrolled. The mean age was 54.8 ± 9 years, and the mean body mass index was 26.5 ± 3.6. The overall prevalence of ED in the study population was 59.5% (119 patients) (CI 95%: 52%–67%). The mean score of potency in the impotent group was 11.1 ± 6.4. Age, BMI, systolic blood pressure (SBP), and creatnin (Cr) were significantly different between ED positive group and ED negative group. Age (*P* < 0.001), SBP (*P*: 0.02), and Cr level (0.04) was significantly higher in ED positive group. In contrast, BMI (*P*: 0.01) was considerably lower in ED positive patients in compare with the ED negative one. The demographic characteristics of the study population, and the comparison between ED positive and ED negative group were shown in Table 1. The prevalence of ED increased with increasing age; men aged >60 years had a prevalence of 77.1% compared to 34.8% among men aged <50 years (*P*: 0.001). 

Potency score in patients had significant correlation with SBP, FPG, HbA1c, and creatinin concentration but not with LDL, HDL, TG, BMI, and serum testosterone (Table 2). 

Logistic regression analysis was employed to predict the probability of ED and parameters which influenced that. The predictor variables were participants' age, HbA1c, SBP, DBP, BMI, creatinin, duration of diabetes, and using calcium channel blocker.  Table 3 shows the logistic regression coefficients, Wald test and odds ratio with 95% confidence interval for each of the predictors. Employing a 0.05 criterion of statistical significance, age, and use of calcium channel blocker, but no other variable had significant partial effects. The significant odds ratios for older age and taking CCB medication were calculated while holding all other variables constant. Older participants were 2.8 times (*P*: 0.017), and patients taking CCB medication were 4.1 times (*P*: 0.01) more likely to have ED (Table 3).

## 4. Discussion

We found that age and taking CCB medication in T2DM men were the only independent predictors and other previously published risk factors including BMI, SBP, DBP, HbA1c, impaired lipid profile, high creatinin, testosterone level, and even history of smoking did not have predictive value for ED risk in T2DM patients.

The prevalence of erectile dysfunction among diabetic men which had been reported previously varies between 35 and 90% [11–15]. For instance, erectile dysfunction was detected in over 50% of men with diabetes in the U.S [16] and in 41% of diabetic men in the Netherlands [17]. Studies from Saudi diabetic patients reported ED among 80 to 90% of the patients [18, 19]. There is a limited data regarding ED prevalence in diabetic patients in Iran. In a recent study sexual dysfunction was detected in 77% of diabetic men in Isfahan province of Iran [20]. In our study a significant number of patients had ED (59%). The higher prevalence reported by some studies could be explained by the fact that they did not consider any psychological factors affecting ED in diabetes which can passively increase ED in these patients. In the present study, we have excluded these confounding factors to eliminate unreal data. In addition the higher prevalence of ED reported in these kind of studies may be because of selection bias of referral centers, as most of the studies were undertaken in referral centers and their study population may contain more complicated diabetic patients. We used more general setting for the study to make better external validity for the results.

HbA1c, duration of diabetes, blood pressure, and use of diuretics were adjusted for, but were not significant in the model and so could not independently predict ED in diabetic patients of our study. On the contrary, longer duration of DM and poorer glycemic control in diabetic men have been reported by some studies previously as the predictors of ED in DM [2, 3, 5, 11, 21] Lu et al. in a study concluded younger men with T2DM probably give more benefits of better glycemic control rather than older diabetic men to reduce the prevalence of ED [22]. In our study, we found only a significant negative correlation between HbA1c and potency score of the subjects. These results revealed that age is the most potent risk factor for the existence of ED in men with DM and the poorer glycemic control related to higher degrees of impotence but it is not an independent risk for ED. Although most studies have been able to adjust their results for age, only a few have had sufficiently robust data to adjust simultaneously for age, diabetes control, and other risk factors.

Surprisingly men with ED had lower BMI in our study that we found no study with similar result. Previous studies have reported no correlation between ED and BMI [20, 23]. In support, with using score of potency instead of existence of impotence, no correlation was found between BMI and erectile function of diabetic men in our study.

Important cardiovascular risk factors and metabolic syndrome elements, which were considered in this study including FPG, blood pressure, atherogenic dyslipidemia (low levels of HDL and high levels of triglycerides) were not associated with ED in T2DM patients, whereas SBP and FPG showed a negative correlation with potency score.

Over the past two decades, there have been many studies reported intimate association among ED and obesity, dyslipidemia, T2DM, HTN, metabolic syndrome and CVD. Many of them believe these metabolic and vascular conditions share important risk factors. Some prospective [24, 25] and retrospective [26] studies have shown ED as an independent risk factor for and precursor to CVD, and high prevalence of ED in patients with CVD. Whereas others have revealed patients with ED have similar prevalence of CVD risk factors in compare with general population [27]. The mechanism underlying the relation between ED and these metabolic conditions is not clearly known. Many etiologies for ED in DM were presented; some of them could be probable link between ED and CVD. Endothelial dysfunction, diabetic vasculopathy and neuropathy, dyslipidemia, hypogonadism and psychological disorders that were commonly found in diabetics have been suggested as pathophysiology of ED in diabetic patients [28].

A recent study [29] showed prevalence of penile arterial lesions is very rare in patients with coronary and peripheral arterial atherosclerosis which attenuate the hypothesis of penile atherosclerosis as a link between ED and CVD. Whereas they revealed diabetes as only factor associated with penile atherosclerosis. In addition some experimental studies exhibited that erectile function were significantly damaged in hyperlipidemic conditions because of some structural change of penile vessels [30, 31]. Our study did not show any relation between impaired lipid profile or hypertension, the known risk factors of atherosclerosis, and ED in patients with T2DM.

Finally we were encountered with some limitations; one of them was psychological evaluation of the patients. Although patients with major depression and other overt psychological disorders were excluded from our study, detailed psychological evaluation could not be considered for them.

## 5. Conclusion

In conclusion, we found that ED prevalence is high in men with T2DM and is associated with some variables, most notably with age and taking CCB. Other metabolic and sexual covariates in multivariate regression analysis model fall short of significance in our study so despite many previous evidence of relation among lipid profile, glycemic control, duration of DM, HTN and testosterone level and ED in T2DM, we did not come to the same conclusion.

## Figures and Tables

**Table 1:** Clinical, biochemical, and hormonal characteristics of men with type 2 diabetes according to their sexual function (*n* = 200).

Variable	Men with normal sexual function (*n* = 81)	Men with sexual dysfunction (*n* = 119)	*P* value	All the participants (*n* = 200)
Age (year)	51.7 ± 8.9	57.3 ± 9.2	<0.001	54.8 ± 9.4
Diabetes duration (Month)^∗^	6.21 ± 3.9	7.67 ± 5.3	0.1	7.01 ± 4.7
Smoking (%)	16.4%	22.2%	0.37	19%
BMI (Kg/m^2^)	27.3 ± 3.6	25.9 ± 3.4	0.01	26.5 ± 3.6
SBP (mmHg)	127.4 ± 16.1	133.9 ± 18.5	0.02	130 ± 17
DBP (mmHg)	80.4 ± 9	82 ± 9.3	0.2	81 ± 9
FPG (mg/dL)	135.7 ± 53.2	143.7 ± 53.6	0.3	140 ± 53
TG (mg/dL)^∗^	189.9 ± 140.6	187.3 ± 70.1	0.2	183.5 ± 110.5
Chol (mg/dL)	177.8 ± 35.3	181.5 ± 36	0.5	179 ± 36
HDL-C (mg/dL)^∗^	39.5 ± 7.8	39.5 ± 5.2	0.8	39.5 ± 6.5
HbA1c (%)	7.7 ± 1.8	7.7 ± 1.4	0.8	7.7 ± 1.6
Creat (mg/dL)^∗^	1.09 ± 0.21	1.16 ± 0.23	0.04	1.13 ± 0.22
Testosterone (ng/dL)^∗^	6.04 ± 2.5	5.9 ± 1.9	0.8	5.9 ± 2.2

BMI: body mass index, SBP: systolic blood pressure, DBP: diastolic blood pressure, FPG: fasting plasma glucose, Tg: triglyceride, Chol: cholesterol, HDL-C: high density lipoprotein cholesterol, Creat: creatinin concentration.

^∗^Because of abnormal distribution of these variables we tested them with nonparameteric Mann-whitney test but no difference was found in the results and prevalues.

**Table 2:** Correlation between potency score and quantitative variables in men with type 2 diabetes (*n* = 200).

Variable	Potency score	
	Correlation	*P* value
Age	−0.44	<0.001^∗^
BMI	0.002	0.9
SBP	−0.18	0.02^∗^
DBP	−0.07	0.4
Hb A1c	−0.2	0.01^∗^
FPG	−0.17	0.03^∗^
TG	−0.12	0.1
Chol	−0.01	0.8
HDL-C	−0.01	0.8
Creat	−0.2	0.014^∗^
Testosterone	0.11	0.16

BMI: Body Mass Index, SBP: systolic blood pressure, DBP: diastolic blood pressure, FPG: fasting plasma glucose, Tg: triglyceride, Chol: cholesterol, HDL-C: high density lipoprotein cholesterol, Creat: creatinin concentration.

^∗^Correlation is significant at the 0.05 level.

**Table 3:** Logistic regression analysis of confounding variables to predict sexual dysfunction in men with type 2 diabetes mellitus (*n* = 200).

Dependent variable	Independent variables	Odds ratio	*P* value
	Age	2.8	0.015
	Diabetes duration	1	0.4
	HbA1c	1	0.5
Sexual dysfunction	SBP	0.47	1
	BMI	0.9	0.06
	Use of diuretic	1.9	0.3
	Use of CCB	4.1	0.01
	Creat	1.4	0.7

BMI: body mass index, SBP: systolic blood pressure, FPG: fasting plasma glucose, CCB: calcium chanel blocker, Creat: creatinin.
